# Salt stress responses and SNP‐based phylogenetic analysis of Thai rice cultivars

**DOI:** 10.1002/tpg2.20189

**Published:** 2022-01-07

**Authors:** Susinya Habila, Nopphakhun Khunpolwattana, Thanin Chantarachot, Teerapong Buaboocha, Luca Comai, Supachitra Chadchawan, Monnat Pongpanich

**Affiliations:** ^1^ Center of Excellence in Environment and Plant Physiology, Dep. of Botany, Faculty of Science Chulalongkorn Univ., Pathum Wan District Bangkok 10330 Thailand; ^2^ Dep. of Plant Science and Biotechnology, Faculty of Natural Science Univ. of Jos, Jos Plateau State Nigeria; ^3^ Molecular Crop Research Unit, Dep. of Biochemistry, Faculty of Science Chulalongkorn Univ., Pathum Wan District Bangkok 10330 Thailand; ^4^ Omics Sciences Center, Faculty of Science Chulalongkorn Univ., Pathum Wan District Bangkok 10330 Thailand; ^5^ Genome Center and Dep. of Plant Biology UC Davis Genome Center, Univ. of California–Davis Davis CA 95616 USA; ^6^ Dep. of Mathematics and Computer Science, Faculty Science Chulalongkorn Univ., Pathum Wan District Bangkok 10330 Thailand

## Abstract

Genetic diversity is important for developing salt‐tolerant rice (*Oryza sativa* L.) cultivars. Certain Thai rice accessions display salt tolerance at the adult or reproductive stage, but their response to salinity at the seedling stage is unknown. In this study, a total of 10 rice cultivars/line, including eight Thai cultivars and standard salt‐tolerant cultivar and susceptible line, were screened using a hydroponic system to identify salt‐tolerant genotypes at the seedling stage. Different morphophysiological indicators were used to classify tolerant and susceptible genotypes. Phylogenetic analyses were performed to determine the evolutionary relationships between the cultivars. Results showed that ‘Lai Mahk’, ‘Jao Khao’, ‘Luang Pratahn’, and ‘Ma Gawk’ exhibited salt stress tolerance comparable with the standard salt‐tolerance check ‘Pokkali’. Whole‐exome single‐nucleotide polymorphism (SNP)‐based phylogenetic analysis showed that the Thai rice cultivars were monophyletic and distantly related to Pokkali and IR29. Lai Mahk and Luang Pratahn were found closely related when using the whole‐exome SNPs for the analysis. This is also the case for the analysis of SNPs from 164 salt‐tolerance genes and transcription regulatory genes. The tolerant cultivars shared the same haplotype for 16 genes. Overall, the findings of this study identified four salt‐stress‐tolerant Thai rice cultivars, which could be used in rice breeding programs for salinity tolerance.

## Core Ideas

- Salinity tolerance is an important trait for sustainable rice production.
- SNPs from 164 salt‐tolerant genes were identified in salt‐tolerant cultivars.
- ‘Jao Khao’, ‘Lai Mahk’, and ‘Luang Pratahn’ could be used for breeding salinity tolerant rice.

**Abbreviations**
CMS: cell membrane stability
QTL: quantitative trait loci
RDW: root dry weight
RFW: root fresh weight
RIL: recombinant inbred line
RL: root length
RWC: relative water content
SDW: shoot dry weight
SES: standard salt injury evaluation system
SFW: shoot fresh weight
SI: stability index
SL: shoot length
SNP: single‐nucleotide polymorphism

## 1 INTRODUCTION

Rice (*Oryza sativa* L.) is one of the most salt‐sensitive glycophytic cereals because of its inability to regulate Na^+^ influx through the root (Reddy et al., 2017), leading to rapid accumulation of cytotoxic Na^+^ in the shoots, thereby affecting growth and yield. Under salt stress, leaf temperature increases, and cell elongation is inhibited by stomatal closure (Rajendran et al., 2009; Sirault et al., 2009). Moreover, salinity can inhibit seed germination (Hakim et al., 2010; Shobbar et al., 2012) and cause chloroplast destruction (Yamane et al., 2008), leading to an overall decrease in photosynthesis (Moradi & Ismail, 2007). Studies have shown that rice seedlings are highly susceptible to salt stress. Salinity tolerance increases with age and development until early reproductive stage, when rice plants become susceptible again (Pearson et al., 1966). Salt tolerance is a polygenic trait that involves multiple cellular pathways and mechanisms. Quantitative trait loci (QTL) for salt tolerance in rice have been identified using different techniques at different developmental stages such as QTL mapping at the seedling stage (Ghomi et al., 2013), genome‐wide association studies at the flowering stage, yield components (Lekklar et al., 2019a; Mohammadi et al., 2013), and the combination of QTL mapping and transcriptome profiling of bulk recombinant inbred lines (RILs) (Pandit et al., 2010).

Several genes related to salt‐stress response and salt tolerance have been identified in rice including genes involved in transport processes, signaling, and antioxidative response (Reddy et al., 2017). The ion transport process is important for ion homeostasis under salt‐stress conditions. Furthermore, studies have shown that salt stress causes oxidative stress in plants (Ahmad et al., 2019; Rasool et al., 2013), and enzymes involved in antioxidant activities under stress conditions have been examined. Superoxide dismutase and its chaperones are reported to be involved in the detoxification of cells under stress conditions (Kaminaka et al., 1999). Moreover, there is an increase in ascorbate peroxidase and peroxidase activities in salt‐tolerant rice CSSL16, which is a chromosome substitution line of ‘KDML105’ rice (Chutimanukul et al., 2019).

Cultivated rice displays high genetic variability, which could be important in developing salt‐tolerant rice cultivars. Several researchers have studied the variations in rice species and populations in relation to salinity tolerance (De Leon et al., 2015; Kanawapee et al., 2011; Tin et al., 2021). The salt‐tolerant rice, ‘Pokkali’, and other cultivars have been used for developing salt tolerant cultivars. Gregorio (1997) identified QTL linked with salt tolerance at the seedling stage in RIL populations developed from IR29 and Pokkali. To further understand the genetic mechanism of salinity tolerance in Pokkali, a total of 38 tolerant and 42 sensitive RILs were genotyped using the amplified fragment length polymorphism method, and a significant QTL linked to the Na^+^/K^+^ ratio was discovered on chromosome 1 (Gregorio, 1997). After integrating the restriction fragment length polymorphism and simple sequence repeat markers into the *Saltol* QTL, Bonilla et al. (2002) found a 43% variance in the Na^+^/K^+^ ratio in a 58‐RIL population, resulting in the identification of a highly tolerant RIL, FL478 (IR 66946‐3R‐178‐1‐1) that is used in breeding programs (Thomson et al., 2010). Other salt‐tolerant landraces include the *indica* landrace Nona Bokra from West Bengal (Batayeva et al., 2018; Lin et al., 2004; Waziri et al., 2016) and Horkuch from Bangladesh (Lisa et al., 2004; Razzaque et al., 2019). Recently, ‘Pusa Basmati 1509’, ‘Pusa 44’, and ‘Sarjoo 52’ were improved by the introgression of the *Saltol* QTL from FL478 (Krishnamurthy et al., 2020; Yadav et al., 2020). Although Pokkali or its derivatives could be used in developing salt‐tolerant cultivars, it is preferable and easier to develop novel salt‐tolerant cultivars using local cultivars rather than distant donors. For instance, after evaluating 71 genotypes obtained from the coastal regions of Gao and Karnataka in India, Manohara et al. (2021) identified 10 salinity‐tolerant genotypes at the seedling stage, which might be alternatives to salt‐tolerant donors such as Pokkali and FL478. Haplotype analysis showed that the allelic component of the *Saltol* QTL of the 10 salt‐tolerant genotypes differed from that of the standard check FL478 (Manohara et al., 2021).

**Core Ideas**

- Salinity tolerance is an important trait for sustainable rice production.
- SNPs from 164 salt‐tolerant genes were identified in salt‐tolerant cultivars.
- ‘Jao Khao’, ‘Lai Mahk’, and ‘Luang Pratahn’ could be used for breeding salinity tolerant rice.

Rice is a major crop in Thailand and is grown in all parts of the country; however, some regions are affected by high soil salinity especially the northeastern region where high‐quality rice is produced (Katawatin & Sukchan, 2012). Farmers use seed stocks that are adaptable to their locality, which could help select for salt tolerance traits in some local cultivars. Several studies have examined salt‐stress tolerance in Thai rice (Cha‐um et al., 2009, 2007; Kanawapee et al., 2011, 2013; Kanjoo et al., 2011; Ninsuwan et al., 2013; Nounjan et al., 2016; Pongprayoon et al., 2019). However, most of the studies were limited to a handful of well‐known or improved cultivars, while most of the tested genotypes were inferior to Pokkali in their performance under high salinity. Our previous study explored salt tolerance in a large panel of Thai rice cultivars at the reproductive stage (Lekklar et al., 2019a). The study identified one QTL associated with net photosynthesis at day six under salt stress on chromosome 10, two QTL associated with the number of panicles per plant on chromosomes 5 and 10, one QTL associated with the number of filled grains per plant on chromosome 4, and two QTL associated with the number of unfilled grains per plant on chromosomes 1 and 7 (Lekklar et al., 2019a). Some of the accessions, including ‘Jao Khao’, ‘Luang Pratahn’, and ‘Ma Gawk’, were highly tolerant to salt stress and could potentially be used as donors in breeding programs for the development of novel salt‐tolerant cultivars. However, the performance of these cultivars under salt stress conditions at the seedling stage is unknown. Therefore, the aim of this study was to examine the growth performance, physical damage, and cellular status of eight Thai rice cultivars exposed to salt stress at the seedling stage. Additionally, the phylogenetic relationship between the cultivars was examined using both genome‐wide SNPs and SNPs anchored on selected gene classes. Here we describe the salt‐tolerance phenotype of these Thai cultivars as well as their phylogenetic relationship. The data are consistent with a diverse genetic basis for tolerance, a finding important for future selection of rice cultivars for salt‐tolerant breeding programs.

## 2 MATERIALS AND METHODS

### 2.1 Plant materials

Seeds of eight local Thai rice cultivars, KDML105, Ma Gawk, Jao Khao, ‘Mayom’, ‘Plah Khaeng’, ‘Gam Feuang’, Luang Pratahn and ‘Lai Mahk’, were provided by the Rice Gene Bank of Thailand, Rice Department, Ministry of Agriculture and Co‐operation, Thailand. Seeds of the standard salt‐tolerant cultivar, Pokkali, and the standard salt susceptible line, IR29, were provided by Nakorn Ratchasima Rice Research Center, Thailand.

### 2.2 Evaluation of rice phenotypes after salt‐stress treatment

Salt‐stress responses were evaluated during the seedling stage. The experiment was performed in a randomized complete block design with four biological replicates, comprising the 9 cultivars and 1 line used in this study grown under salt stress and normal conditions. Each replicate contained four plants per cultivar or line. Rice seedlings were grown in a hydroponic system containing a Wagner and Poesh solution (Udomchalothorn et al., 2014). The Wagner and Poesh nutrient solution consists of the following: potassium nitrate (KNO_3_), calcium sulfate (CaSO_4_), magnesium sulfate heptahydrate (MgSO_4_.7H_2_O), triple superphosphate‐monocalcium phosphate Ca(H_2_PO_4_)_2,_ ammonium nitrate (NH_4_)_2_NO_3_, unilate (magnesium oxide‐3.97%; iron‐1.5%; manganese‐1.5%; copper‐0.5%, zinc‐0.5%; boron‐0.3% and molybdenum‐0.026%), and iron ethylenediaminetetraacetic acid. Fourteen‐day‐old seedlings were exposed to salt stress by adding NaCl to the nutrient solution in a stepwise manner until a salinity of 12 dSm^−1^ (100 mM NaCl) was attained after 6 d, and the condition was maintained for another 6 d. Seedlings in the control group were grown in a nutrient solution without NaCl.

Plant phenotypes, including shoot fresh weight (SFW), shoot dry weight (SDW), root fresh weight (RFW), root dry weight (RDW), shoot length (SL), root length (RL), cell membrane stability (CMS), relative water content (RWC), and standard salt injury evaluation system (SES), were evaluated at 3, 6, 9, and 12 d after treatment. Cell membrane stability was determined according to the method of Naghashzadeh (2014), while RWC was determined using the youngest fully expanded leaf according to the method of Sade et al. (2015). Data obtained were all converted into stress susceptibility indices for the phenotypes evaluated according to Fischer and Maurer (1978) by dividing the performances of the cultivars under salt stress by their performances under control conditions. The SES was evaluated according to the method of Gregorio et al. (1997) and was scored in the range of 1‐9, with 1 for the healthy phenotype and 9 for the total death of the plant.

### 2.3 Phylogenetic analysis of salt tolerant genes

The whole‐exome DNA sequences of the 10 rice cultivars/line were obtained from the Omics Sciences and Bioinformatics Center, Faculty of Science, Chulalongkorn University (Lekklar et al., 2019a). The bioinformatics tools GATK haplotype caller (Auwera et al., 2013), FREEBAYES (Garrison & Marth, 2012), and VARSCAN (Koboldt et al., 2013) were used to call variants of each rice cultivar/line. Positions matched by at least two out of the three callers were obtained using vcf‐isec (Danecek et al., 2011) and annotated using the vcf‐annotate tool (Danecek et al., 2011). 

To obtain characterized salt‐tolerance genes from the rice genome, a total of 6,000 corpuses that contained the terms ‘salt tolerance’ or ‘salinity tolerance’ together with ‘rice’ in the title or the abstract were downloaded from Semantic Scholar; only those with PubMed ID were retained, resulting in 621 papers. A parallel PubMed search was performed using the keywords ‘salt tolerance’ and ‘rice’ to search for abstracts containing the desired information, resulting in 511 papers. The papers obtained from the Semantic Scholar and PubMed searches were combined. A BioReader web service (https://services.healthtech.dtu.dk/service.php?BioReader‐1.2) was used to classify the articles into two categories: relevant and irrelevant. To this end, we used PubMed IDs of manually curated relevant articles (positive set, 73 articles) and irrelevant articles (negative set, 73 articles) for training. The results from the BioReader yielded 212 relevant articles. Only those with characterized salt‐tolerant genes were chosen for phylogenetic analyses. As a result, a total of 108 genes were extracted from the 212 ‘relevant articles’ category. The 108 salt‐tolerance‐associated genes were combined with genes with the keywords ‘salt tolerance’ and ‘salt sensitivity’ from an online database (https://funricegenes.github.io/), resulting in 164 genes (Supplemental Tables 1 and 2).

Gene ontology enrichment analysis was carried out on a set of 164 genes (Supplemental Table 3) using R software (Backman & Girke, 2016). Genes that belonged to gene ontology terms associated with gene regulation were grouped. Then, SNPs in this group of genes were concatenated for each cultivar/line into a PHYLIP alignment file (.phy). Heterozygous SNPs were excluded because they could be from pseudogene regions. Phylogenetic analyses based on the created PHYLIP files were carried out using IQ‐TREE v2.1.2 (Minh et al., 2020). We used ModelFinder, which chose the model that minimizes the Bayesian information criterion score, applied an ascertainment bias correction model, and ran an ultrafast bootstrap for 10,000 replicates. The command used was iqtree ‐s input.phy ‐m MFP+ASC ‐bb 10000 ‐seed 1701, where MFP is a model finder, ASC is an ascertainment bias correction, and bb is an ultrafast bootstrap approximation. We used iTOL (Interactive Tree of Life), an online tool to visualize the trees.

### 2.4 Salt‐tolerant gene haplotype and clustering analysis in Thai cultivars

For each gene, the function ghap.haplotyping from the R package GHap was used to call haplotypes from SNP data (Utsunomiya et al., 2016). For visualization, a haplotype network was created using Population Analysis with Reticulate Trees (PopART) with minimum spanning network inference method for each gene (Bandelt et al., 1999).

The number of nucleotide differences in each gene were calculated for all pairwise cultivars/line. Next, a pairwise dissimilarity matrix was computed based on nucleotide differences using the Euclidean distance. Finally, agglomerative hierarchical clustering based on the dissimilarity matrix was performed for all 101 genes and for a set of genes whose haplotype was associated with tolerance, that is, haplotypes shared among the tolerant and sensitive cultivars/line, using hclust function with the agglomeration method ‘ward.D2’ in R.

### 2.5 Statistical analysis

To evaluate differences in stress stability indices for each trait, analysis of variance (ANOVA) based on general linear model and comparison of means by Duncan's multiple range test were conducted using SPSS Statistics 20 software. The cultivars/line were considered fixed factors, whereas each trait was a dependable variable. The ANOVA results were considered significant at *p* < .05.

## 3 RESULTS

### 3.1 Ten rice cultivars/line vary in their growth response to salt stress

**FIGURE 1:**
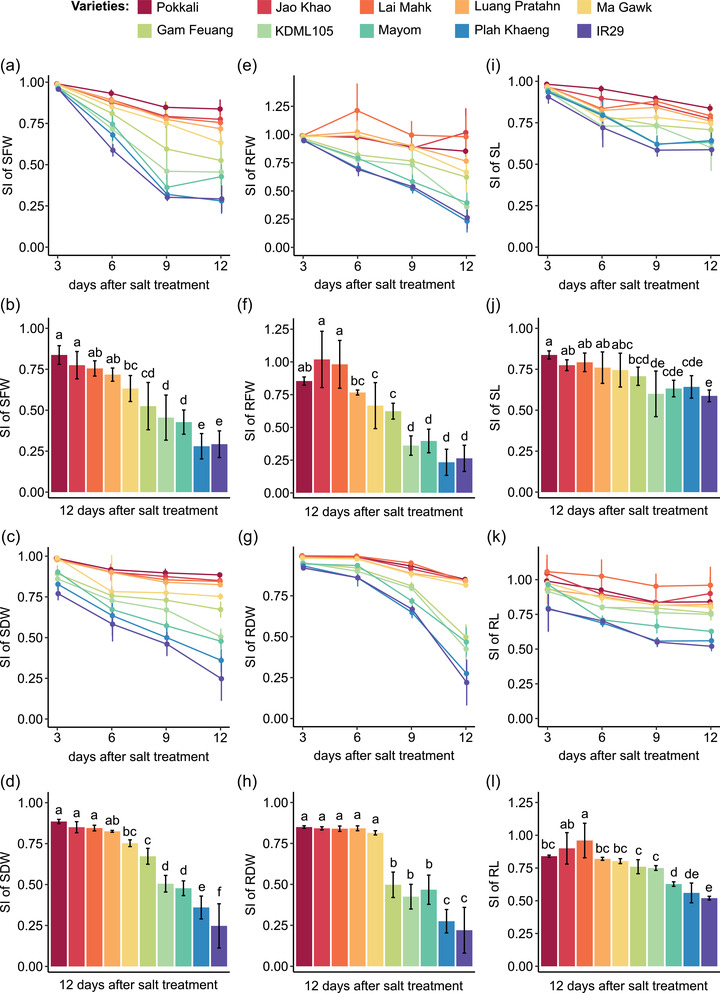
The mean stability indices (SIs) of the growth parameters for the Thai rice cultivars under salt stress. (a, b) Shoot fresh weight (SFW) (g); (c, d) shoot dry weight (SDW) (g); (e, f) root fresh weight (RFW) (g); (g, h) root dry weight (RDW) (g); (i, j) shoot length (SL) (cm); (k, l) root length (RL) (cm). The bar represents the standard error of four replicates. The alphabet represents the mean values, and the cultivars/line having the same alphabet are not significantly (*P* ≤ .05) different based on the Duncan's multiple range test

Results describing the effect of salt stress on growth rate showed that Pokkali, Jao Khao, Lai Mahk, Luang Pratahn, and Ma Gawk displayed better growth than IR29, Plah Khaeng, KDML105, Gam Feuang, and Mayom under salt‐stress conditions. The stability indices (SI) of SFW and SDW of Pokkali were highest but not significantly different from those of Jao Khao, Lai Mahk, and Luang Pratahn, which were higher than 0.7 and 0.8, respectively, after 12 d of salt stress. Additionally, the SI of SFW and SDW of IR29, Plah Khaeng, KDML105, Gam Feuang, and Mayom decreased by >60% under salt stress condition (Figure 1a–d).

The RFW (Figure 1e,f) and RDW (Figure 1g,h) of IR29, Plah Khaeng, KDML105, Gam Feuang, and Mayom decreased to approximately 40% after 12 d of salt stress. Interestingly, Lai Mahk had an SI of RFW of >1 after 6 d of salt stress, indicating an increase in RFW under salt stress compared with that of plants grown under normal conditions. Additionally, compared with other cultivars, the RFW of Lai Mahk and Jao Khao were not significantly affected after 12 d of salt stress (Figure 1f). However, there was a significant decrease in the RDW of all cultivars tested after 12 d of salt stress (Figure 1g,h). Based on percentage decrease in RDW, the cultivars were classified into three categories: Pokkali, Jao Khao, Lai Mahk, Luang Pratahn, and Ma Gawk (10–20% decrease); KDML105, Gam Feuang, and Mayom (50–60% decrease); and IR29 and Plah Khaeng, (>70% decrease) (Figure 1h).

Shoot length (Figure 1i,j) and RL (Figure 1k,l) were significantly correlated with SFW, SDW, RFW, and RDW. Among the parameters examined, the height and SL of Pokkali seedling were least affected by salt stress; however, the differences were not significant. There was a 16 and 40% decrease in the SL of Pokkali and IR29, respectively, indicating that IR29 was the most sensitive line to salt stress (Figure 1j). Additionally, compared with seedlings grown under normal conditions, salt stress caused a 20–26% decrease in the SL of Jao Khao, Lai Mahk, Luang Pratahn, and Ma Gawk and a 29–40% decrease in the SL of KDML105, Gam Feuang, and Mayom. Regarding RL, Lai Mahk was the least affected by salt stress after 12 d, with only 4% decrease in RL compared with that of seedlings grown under normal conditions. In contrast, the RL of Pokkali and IR29 decreased by 16 and 48%, respectively under salt stress conditions, indicating that IR29 was most susceptible to salt stress (Figure 1l).

### 3.2 Salt‐stress‐induced cellular responses correlates with the growth performance

**FIGURE 2:**
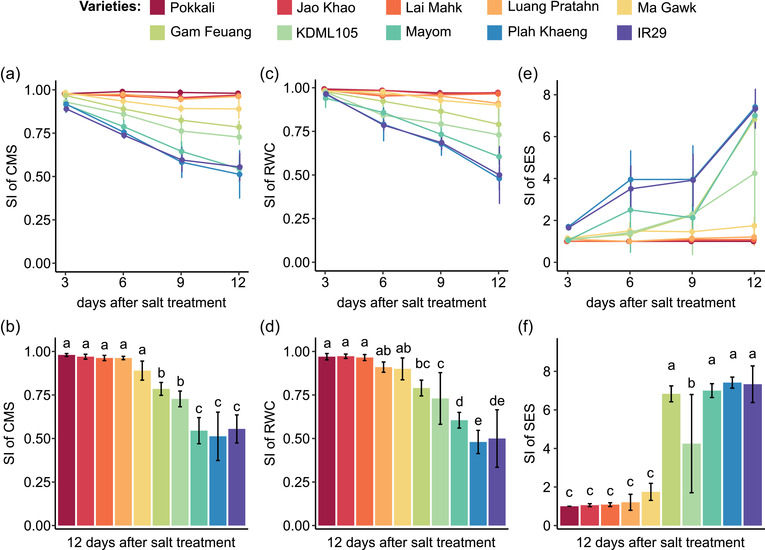
The mean stability indices of the salt responsive traits for Thai rice cultivars under salt stress conditions. (a, b) Cell membrane stability (CMS); (c, d) relative water content (RWC); (e, f) standard salt injury evaluation system (SES). The bar represents the standard error of four replicates. The alphabet represents the mean values, and the cultivars/line having the same alphabet are not significantly (*P* ≤ .05) different based on the Duncan's multiple range test

To determine the cellular status of the cultivars/line under salt stress conditions, the CMS and RWC of the seedlings were examined after 12 d of salt stress. Results showed that the CMS values of Pokkali, Jao Khao, Lai Mahk, Ma Gawk, and Luang Pratahn were not significantly different after 12 d of salt stress (Figure 2a,b), with values ranging from 89–95%. In contrast, there was a significant decrease (50%) in the CMS of IR29, Plah Khaeng, and Mayom. Furthermore, the CMS values of KDML105 and Gam Feuang were 73 and 79%, respectively. A similar pattern was observed for the RWC of the cultivars/line (Figure 2c,d). Based on these two parameters, Pokkali was the most salt‐stress‐tolerant cultivars, whereas Plah Khaeng was the most susceptible.

The measurement of the overall aboveground phenotypes using the SES showed that Pokkali had no visible injury symptoms, with an SES of 1 (Figure 2e,f). Similarly, Jao Khao, Lai Mahk, and Luang Pratahn were resistant to salt stress, with SES values ranging from 1.1 to 1.2 after 12 d of salt stress. In contrast, the remaining cultivars, except Ma Gawk and KDML105 (SES values of 1.7 and 4.3, respectively), were adversely affected by salt stress, with SES values >6.0 (Figure 2e,f).

**FIGURE 3:**
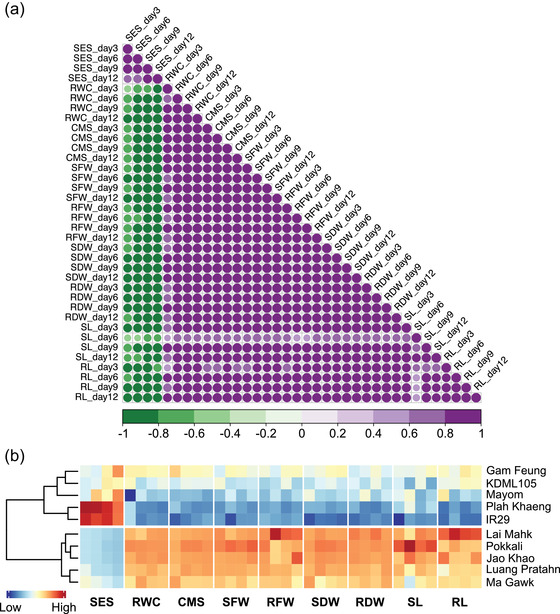
(a) Correlational plot for the stability indices (SIs) of all parameters measured and (b) Hierarchical clustering analysis of the Euclidian distances between the stability indices for all the phenotypes of Thai rice cultivars evaluated under salt stress condition. SES, standard salt injury evaluation system; CMS, cell membrane stability; RWC, relative water content; SFW, shoot fresh weight; RFW, root fresh weight; SDW, shoot dry weight; RDW, root dry weight; SL, shoot length; RL, root length

Furthermore, the salt‐responsive phenotypes evaluated were significantly positively correlated (0.6311–0.9959), except SES, which was significantly negatively correlated (−0.9644 to −0.601) with the other parameters at all time points (Figure 3a; Supplemental Table S4). Hierarchical clustering analysis of the phenotypic data categorized the rice cultivars/line into two major clusters (Figure 3b). The first cluster contained Lai Mahk, Pokkali, Jao Khao, Luang Pratahn, and Ma Gawk, whereas the second cluster contained Gam Feuang, KDML105, Mayom, Plah Khaeng, and IR29 (Figure 3b). Overall, these results indicated the varying degrees of salt tolerance in Thai rice accessions and identified Jao Khao, Lai Mahk, Luang Pratahn, and Ma Gawk as salt tolerant cultivars at the seedling stage.

### 3.3 Whole‐genome SNP‐based phylogenetic analysis separated the standard tolerant and susceptible checks from the Thai rice accessions

**FIGURE 4:**
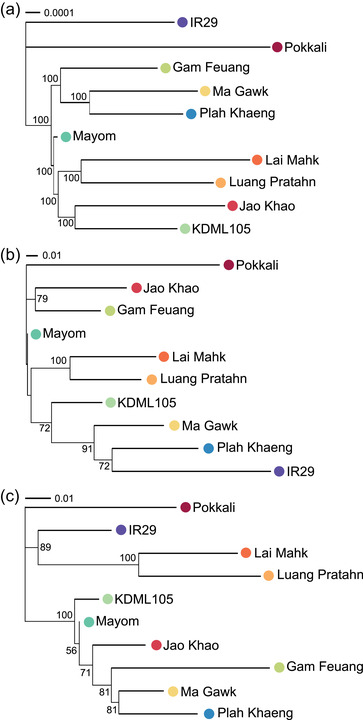
Phylogenetic tree of the cultivars/line based on (a) whole‐genome single‐nucleotide polymorphism (SNP), (b) SNPs from 164 salt‐tolerant genes characterized in rice and other plants, and (c) SNPs from genes encoding transcription factors. The degree of salt tolerance is represented by color, from very tolerant (dark red) to sensitive (blue)

To determine the genetic kinship and variation between the rice cultivars/line examined, we performed whole‐genome SNP‐based phylogenetic analysis using 995,523 SNPs identified in the 10 cultivars/line. The phylogenetic tree showed that the eight local Thai rice cultivars were monophyletic and therefore distinct from Pokkali and IR29 (Figure 4a). Among the Thai rice accessions, three out of the four salt‐tolerant cultivars, including Jao Khao, Lai Mahk, and Luang Pratahn, were clustered in the same clade with the salt‐sensitive cultivar KDML105 (Figure 4a). Overall, these four cultivars appeared to share an ancestor with Mayom. Furthermore, the salt‐tolerant cultivar Ma Gawk was clustered with Plah Khaeng and Gam Feuang, which were both sensitive to salt stress (Figure 4a).

### 3.4 Genetic composition of salinity tolerance in Thai rice

To explore the potential genetic makeup of salinity tolerance in Thai rice, we performed a phylogenetic analysis using 888 SNPs obtained from 164 salt‐tolerant genes reported in rice (Supplemental Table S1 and S2). In contrast to the results of the whole‐exome SNP‐based phylogenetic analysis, phylogenetic analysis using SNPs from salt‐tolerant genes showed that IR29 was closely related to Plah Khaeng, which was highly sensitive to salt stress (Figure 4b). Although Pokkali remained an outgroup, there was no apparent separation of the salt‐tolerant Thai rice cultivars from the susceptible cultivars. Like results of the whole‐exome SNP‐based phylogenetic analysis, Lai Mahk and Luang Pratahn appeared to share a more recent ancestor and were related to Jao Khao (Figure 4a). Overall, these results suggest that the genetic basis for salinity tolerance in Lai Mahk and Luang Pratahn is distinct from that of Jao Khao.

### 3.5 SNPs from genes involved in gene regulation support salt tolerance phenotypes in Thai rice

Since genes encoding regulators of gene expression, such as transcription factors, play a crucial role in salt stress response and tolerance, we considered 230 SNPs derived from 55 genes in this category (Supplemental Table S5). Phylogenetic analysis of SNPs of genes involved in gene regulation showed that Lai Mahk and Luang Pratahn were closely related and shared a common ancestor with IR29 (Figure 4c). Similarly, Pokkali was an out group, whereas the remaining Thai rice cultivars were clustered into one clade, comprising of both salt‐tolerant and salt‐sensitive cultivars. Taken together, these results indicated a close genetic relationship between Lai Mahk and Luang Pratahn at both the whole‐exome and salt‐stress specific gene levels. The distance between the Lai Mahk/Luang Pratahn lineage and those of Jao Khao, Ma Gawk, and Pokkali for salt tolerance genes suggest a diversity in genes encoding regulators of gene expression related to salinity tolerance.

### 3.6 Haplotype analysis confirms the genetic diversity of Thai cultivars in salinity tolerance

To understand the salt tolerance diversity observed among the Thai cultivars in this study, we performed a haplotype analysis for each gene. Haplotype calls were obtained for genes with at least two SNPs, resulting in 101 genes. The highest number of haplotypes (8 haplotypes) was found in *OsHAK1* (*Os04g0401700*) and *OsNHX5* (*Os09g0286400*) genes, while *OsHKT8* (*Os01g0307500*) and *OsbHLH2* (*Os11g0523700*) genes had seven haplotypes. Up to 21 genes showed only two haplotypes. Haplotype number and nucleotide sequences of each gene are shown in Supplemental Tables S6 and S7, respectively. The tolerant cultivars (Pokkali, Jao Khao, Lai Mahk, Luang Pratahn, and Ma Gawk) shared the same haplotype for 16 genes (Supplemental Table S8), whereas the sensitive cultivars (Gam Feuang, KDML105, Mayom, Plah Khaeng, and IR29) shared the same haplotype for 26 genes (Supplemental Table S9). There were 18 genes for which the alleles were shared by the tolerant Thai cultivars but not Pokkali (Supplemental Table S10).

Some genes encoding transcription factors displayed haplotypes associated with salt tolerance or salt sensitivity. Five transcription factor genes sharing the same haplotype among the tolerant cultivars were *OsWARK1*, *OsNF‐YC1*, *OsbZIP23*, *OsMYB106*, and *ONAC106*, while six transcription factor genes sharing the same haplotype among the susceptible cultivars/line were *OsDREB1B*, *OsDREB2A*, *OsHsfC1b*, *OsAP2‐125*, *OsSERF1*, and *OsbZIP71*.

**FIGURE 5:**
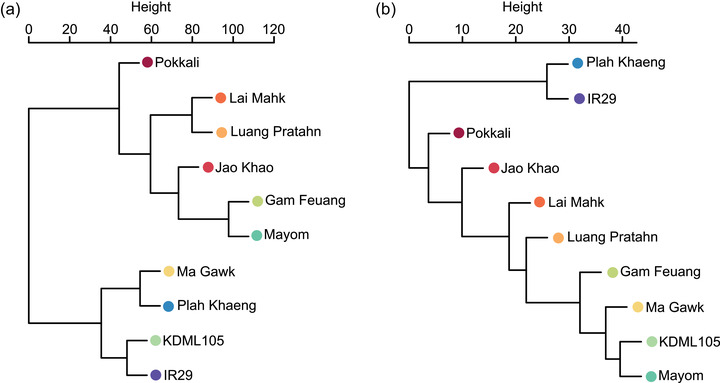
(a) Hierarchical clustering analysis of the Euclidian distances of all 101 salt tolerance genes. (b) Hierarchical clustering analysis of the Euclidian distances of the combination of 16 genes whose alleles were shared by tolerant cultivars and 26 genes whose alleles were shared by sensitive cultivars/line. The degree of salt tolerance is represented by color, from very tolerant (dark red) to sensitive (blue)

A hierarchical clustering analysis of 101 genes separated the cultivars/line into two major groups: the tolerant and the sensitive ones (Figure 5a). The top four tolerant cultivars, Pokkali, Lai Mahk, Luang Pratahn, and Jao Khao, were grouped together. Lai Mahk and Luang Pratahn were clustered together in a subcluster. The three sensitive cultivars/line, Plah Khaeng, KDML105, and IR29, were clustered together along with Ma Gawk (Figure 5a).

By focusing on the 16 genes with shared haplotypes among tolerant cultivars (Supplemental Table S8) and the 26 genes with shared haplotypes among sensitive cultivars/line (Supplemental Table S9), two major clusters were displayed, where Plah Khaeng and IR29 were separated from the others into the first cluster (Figure 5b). In another group, Pokkali was the first to split followed by Jao Khao, Lai Mahk, and Luang Pratahn, where the order of the split corresponded with tolerance phenotype levels.

**FIGURE 6:**
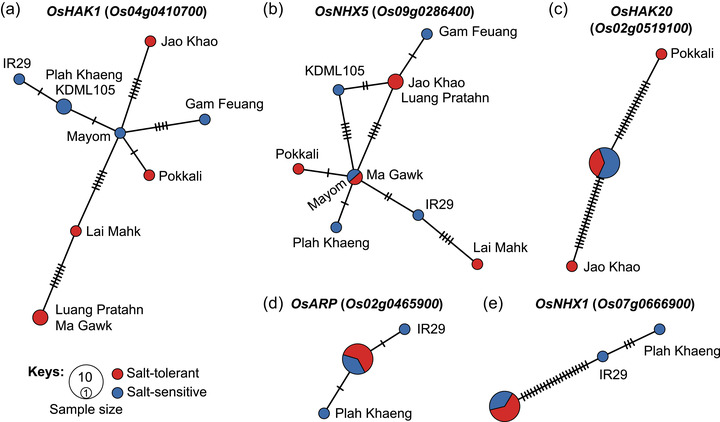
Haplotypes of the transporter genes (a) *OsHAK1*, (b) *OsNHX5*, (c) *OsHAK20*, (d) *OsARP*, and (e) *OsNHX1* visualized by ‘PopART’. The red circles represent the tolerant cultivars, while the blue circles represent the sensitive cultivars/line. The size of the circle represents the number of cultivars having the same haplotypes. The ticks across the graph edges show the number of single‐nucleotide polymorphisms between the connected circles

**FIGURE 7:**
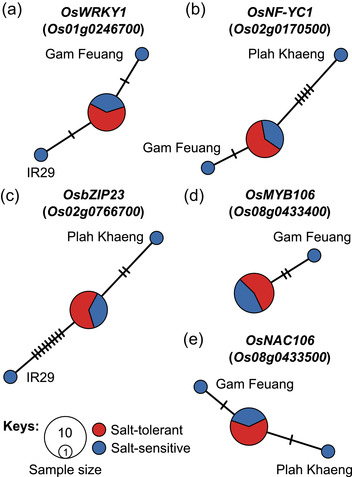
Haplotypes of the transcription factors genes (a) *OsWRKY1*, (b) *OsNF‐YC1*, (c) *OsbZIP23*, (d) *OsMYB106*, and (e) *OsNAC106* visualized by ‘PopART’. The red circles represent the tolerant cultivars, while the blue circles represent the sensitive cultivars/line. The size of the circle represents the number of cultivars having the same haplotypes. The ticks across the graph edges show the number of single‐nucleotide polymorphisms between the connected circles

Specific haplotypes of the salt tolerance genes may enhance salt tolerance. Both Na^+^ and K^+^ transporters have a role in ion homeostasis during salt stress, and they are important for salinity tolerance (Assaha et al., 2017). Based on this analysis, *OsHAK1* and *OsNHX5* showed the highest haplotype variations (Figure 6a). The SNPs in *OsHAK1* of Pokkali, Jao Khao, Lai Mahk, Luang Pratahn, and Ma Gawk deviated from other salt‐susceptible cultivars/line, resulting in the different haplotypes (Figure 6a). Similar deviation of *OsNHX5* haplotypes was also detected (Figure 6b). Many SNPs were detected in *OsHAK20* of Pokkali and Jao Khao (Figure 6c) consistent with the possibility of specialization for salt tolerance in these two cultivars. On the other hand, IR29 and Plah Khaeng showed unique haplotypes in *OsARP*, encoding a vacuolar Na^+^ regulator (Figure 6d), and *OsNHX1*, encoding a Na^+^/H^+^ antiporter (Figure 6e), which may contribute to salt stress susceptibility in these two cultivar/line. Similarly, the salt susceptible cultivars/line, such as IR29, Plah Khaeng, and Gam Feuang, exhibited distinct haplotypes of transcription factor genes including *OsWRKY1*, *OsNF‐YC1*, *OsbZIP23*, *OsMYB106*, and *ONAC106* (Figure 7a−e).

## 4 DISCUSSION

Salt tolerance is a complex trait that is controlled by both environmental (growing conditions and developmental stages) and genetic factors (Reddy et al., 2017). In the present study, the effect of salt stress on the performance of eight Thai rice cultivars was examined at the seedling stage. Results showed that salt stress had varying effects on the growth parameters, visible injury levels, CMS, and RWC of the cultivars. Among the cultivars examined, Pokkali, Jao Khao, Lai Mahk, Luang Pratahn, and Ma Gawk displayed higher levels of salt tolerance compared with the other cultivars, which agreed with the findings of Chokwiwatkul et al. (2017), who reported higher salt‐stress tolerance in the seedlings of Luang Pratahn and Lai Mahk. Similarly, Lekklar et al. (2019b) found that Jao Khao had a significantly higher number of filled grains per plant than other cultivars examined under salt‐stress conditions. In another study, Luang Pratahn and Ma Gawk had higher and similar CMS values, respectively, compared with Pokkali after 9 d of salt stress at the reproductive stage (Lekklar et al., 2019a). Overall, these findings indicate that some Thai rice cultivars possess high salinity tolerance at both the seedling and reproductive stages.

The SES demonstrated that the salt tolerance of Jao Khao, Lai Mahk, Luang Pratahn, and Ma Gawk were comparable with that of Pokkali. In contrast, Plah Khaeng exhibited similar sensitivity to salinity as IR29. Therefore, the salt‐tolerant cultivars are likely better adapted physiologically than the sensitive cultivars. A previous study by Gerona et al. (2019) showed that two salt‐tolerant cultivars, IR670 and Pokkali, had considerably lower Na^+^/K^+^ ratios, higher chlorophyll content, and higher cell integrity under salt stress than sensitive cultivars. Similarly, Singh et al. (2018) stated that the lower Na^+^/K^+^ ratio of the shoots of CSR10 (salt‐tolerant genotype) compared with that of the shoots of MI48 (salt‐sensitive genotype) could be attributed to a decrease in Na^+^ translocation from the roots to the shoots of the tolerant genotype. These findings could explain the improved CMS (low electrolyte leakage), ion homeostasis, and performance of tolerant genotypes compared with sensitive genotypes. In contrast, salt‐sensitive cultivars are characterized by ionic imbalances, altered distribution of nutrients within plants (Islam et al., 2019), and lower net photosynthetic rate, resulting in reduced grain yield after recovery (Lekklar et al., 2019b). In the present study, the higher CMS and RWC observed in Jao Khao, Lai Mahk, Luang Pratahn, and Ma Gawk may be associated with improved Na^+^/K^+^ homeostasis and osmosis. However, the molecular mechanisms underlying these physiological changes associated with the salt‐tolerant phenotypes is poorly understood.

In the present study, a phylogenetic analysis was performed using SNPs obtained from the whole‐exome sequences of the 10 rice cultivars/line (Figure 4a). Results showed the eight local Thai cultivars were distinct from Pokkali and IR29, indicating that the eight cultivars were evolutionarily separated from Pokkali and IR29. This could be attributed to the different geographical origins of Pokkali and IR29 (Ali et al., 2006; Coffman & Gomez, 1978; Krishnamurthy et al., 2014).

Furthermore, in the present study, phylogenetic analysis using 164 salt‐tolerant genes indicated that there were genetic variations in the genes controlling salinity tolerance in the salt‐tolerant cultivars. Further studies are necessary to identify the genes responsible for the salt‐tolerance phenotype in the Thai rice accessions. However, the present study showed that Lai Mahk and Laung Pratahn were closely related, indicating that similar genes may be responsible for salinity tolerance in the two cultivars. Interestingly, Ma Gawk cultivar, which was classified as salt tolerant exhibited the poorest performance among the five tolerant cultivars under salt‐stress conditions. For instance, Ma Gawk had similar SDW to Gam Feuang, a salt‐sensitive cultivar. Additionally, Ma Gawk was grouped in the same clade with Plah Khaeng, IR29, and KDML105. Furthermore, although Lai Mahk and Luang Pratahn share a common ancestor with the sensitive clade (Plah Khaeng, Ma Gawk, IR29, and KDML105), gene transfer or genetic changes may have occurred from selection during domesticated cultivation by farmers, conferring them the salt‐tolerant phenotype. Moreover, the three phylogenetic analyses described in Figure 4 showed that the two cultivars (Lai Mahk and Luang Pratahn) were related, indicating that they may share several traits (Figure 4a–c). Additionally, the findings of the present study showed that Gam Feuang had the best performance among the five sensitive cultivars/line and shared a common ancestor with Jao Khao. Perhaps other salt‐tolerance‐related genes that were not included in the analysis may be responsible for the tolerant phenotype of Jao Khao. Based on the 164 salt tolerance genes’ phylogeny (Figure 4b), the four tolerant Thai cultivars may employ different resistance mechanisms or, at least, use different genes to achieve resistance. Jao Khao mechanisms of resistance may be the closest to those of Pokkali. Lai Mahk and Luang Prathan are more related to each other than to the others, and Ma Gawk is likely to employ the most diverged resistance genes.

Transcriptional regulation of salt tolerance genes likely results in the quantitative changes in critical factors that enable cell survival under environmental stress (Ganie & Reddy, 2021; Ito et al., 2006; Park et al., 2020). Therefore, the roles of transcription regulating gene in salt stress tolerance were examined in the present study, and the results showed that Ma Gawk and Jao Khao were classified in the sensitive clade, like the results of whole‐exome SNP‐based phylogenetic analysis. However, Ma Gawk and Jao Khao were grouped together with KDML105, Mayom, Gam Feuang, and Plah Khaeng but not with IR29. These results indicate that genetic variations across all transcription factors is inadequate to partition the tree into the sensitive and tolerant clades. Additionally, the sensitive Thai cultivars in this group differed in gene regulation from IR29 and Pokkali. On the contrary, Lai Mahk and Luang Pratahn might have similar regulatory mechanisms to Pokkali. Interestingly, IR29 shared a distant common ancestor (long branch) with Lai Mahk and Luang Pratahn, suggesting that different genetic mechanisms underlie salt tolerance in these Thai cultivars. The discrepancy between the genetic distance (Figure 4) and the tolerance phenotype could be because variation affecting different combinations of limited number of genes can impact the ability to cope with salinity. Alternatively, other mechanisms may affect response to salinity, for example, epigenetics, or uncharacterized salt‐tolerance‐related genes were not included in the analysis.

To investigate the sharing of candidate gene haplotypes, we carried out an in‐depth analysis using the R package GHap. Because of difference in geographical origin, it is likely that salt stress tolerance in the Thai cultivars was bred independently from that of Pokkali. The 101 salt‐stress genes clustering analysis, however, revealed a distinct pattern of shared haplotypes (Figure 5a). Likewise, the Thai rice sensitive cultivars shared appreciable haplotypes with IR29. We identified shared haplotypes for 16 genes among the tolerant cultivars and for 26 genes among the sensitive cultivars/line. The clustering analysis from these two gene sets was predictive of their salt‐stress phenotype. First, the two most sensitive were phylogenetically isolated from the rest of the cultivars. Second, the order of the split for the tolerant cultivars (Pokkali followed by Jao Khao, Lai Mahk, and Luang Pratahn) corresponded to the tolerance phenotype level. The individual haplotypes for certain genes were also associated with varietal tolerance (Figure 7). Certain transcription factors are thought to modulate salt tolerance by regulating many salt responsive genes (Guo et al., 2021; Ponce et al., 2021; Shah et al., 2021). ‘Plah Kaheng’, IR29, Gam Feuang, the salt sensitive cultivars/line, showed unique haplotypes in particular transcription factors, suggesting the SNPs in these genes may be directly responsible or linked to variation responsible for salt tolerance in Thai rice cultivars. Ion transporters have the major role in salt tolerance (Shah et al., 2021). For example, the equilibrium of K^+^/Na^+^ ratio is an important determinant in rice salt tolerance (Shah et al., 2021). Based on our investigation, *OsHAK1*, a K^+^ transporter, and *OsNHX5*, a Na^+^ transporter, showed the highest number in haplotypes, indicating high variation in the genes for ion homeostasis in Thai rice population.

In summary, our investigation of haplotypes of salt tolerance genes supported a role for several of them in salt tolerance. In addition, the pattern of haplotype distribution supported the hypothesis that varying combinations of genes can confer salt tolerance, as we did not observe single genes whose haplotypes were completely distinct between the tolerant and sensitive groups. The incongruency of Ma Gawk's phylogenetic pattern also suggested that other genes not included in this study, and potentially mechanisms, could contribute to its salt tolerance phenotype.

## 5 CONCLUSION

In the present study, 10 cultivars/line of rice, including eight local Thai cultivars and the standard salt‐tolerant (Pokkali) and susceptible (IR29) checks were examined to identify potential salt‐tolerant genotypes at the seedling stage. Four Thai rice cultivars, including Lai Mahk, Jao Khao, Luang Pratahn, and Ma Gawk, exhibited similar levels of salt‐stress tolerance as Pokkali. The tolerance phenotype in Jao Khao and Ma Gawk are likely based on different genes and potentially mechanisms. The diversity of these salt‐tolerant cultivars could provide genetic resources useful for breeding broadly based and strong salt‐tolerance into new Thai cultivars.

## AUTHOR CONTRIBUTIONS

Susinya Habila: Conceptualization; Data curation; Formal analysis; Investigation; Writing – original draft. Nopphakhun Khunpolwattana: Data curation; Investigation. Thanin Chantarachot: Formal analysis; Writing – review & editing. Teerapong Buaboocha: Conceptualization; Resources; Supervision. Luca Comai: Writing – review & editing. Supachitra Chadchawan: Conceptualization; Funding acquisition; Resources; Supervision; Writing – review & editing. Monnat Pongpanich: Conceptualization; Formal analysis; Methodology; Supervision; Writing – review & editing.

## CONFLICT OF INTEREST

The authors declare that there are no conflicts of interest.

## Supporting information

Supplemental Table S1: List of salt‐tolerant genes retrieved using the Bioreader data mining tool using PubMed IDs.
Supplemental Table S2: List of salt‐tolerant genes from the Funricegenes database.
Supplemental Table S3. Gene ontology annotation table for all genes.
Supplemental Table S4: Pearson correlation coefficients for all parameters measured at 3, 6, 9, and 12 d after salt stress treatment.
Supplemental Table S5: List of genes involved in gene regulation.
Supplemental Table S6: List of genes showing the kind of haplotype shared by the Thai rice cultivars.
Supplemental Table S7: List of genes showing the number of SNPs and haplotype sequences.
Supplemental Table S8: A list of genes whose alleles are shared by the tolerant cultivars (‘Pokkali’, ‘Jao Khao’, ‘Lai Mahk’, ‘Luang Pratahn’, and ‘Ma Gawk’).
Supplemental Table 9: A list of genes whose alleles are shared by the sensitive cultivars/line (‘Gam Feuang’, ‘KDML105’, ‘Mayom’, ‘Plah Khaeng’, and IR29).
Supplemental Table 10: A list of genes whose alleles are shared by the tolerant Thai cultivars (‘Jao Khao’, ‘Lai Mahk’, ‘Luang Pratahn’) but ‘Pokkali’.

## References

[tpg220189-bib-0001] Ahmad, R. , Hussain, S. , Anjum, M. A. , Khalid, M. F. , Saqib, M. , Zakir, I. , Hassan, A. , Fahad, S. , & Ahmad, S. (2019). Oxidative stress and antioxidant defense mechanisms in plants under salt stress. In M. Hasanuzzaman , K. R. Hakeem , K. Nahar , & H. F. Alharby (Eds.), Plant abiotic stress tolerance: Agronomic, molecular and biotechnological approaches (pp. 191–205). Springer International Publishing.

[tpg220189-bib-0002] Ali, A. J. , Xu, J. L. , Ismail, A. M. , Fu, B. Y. , Vijaykumar, C. H. M. , Gao, Y. M. , Domingo, J. , Maghirang, R. , Yu, S. B. , Gregorio, G. , Yanaghihara, S. , Cohen, M. , Carmen, B. , Mackill, D. , & Li, Z. K. (2006). Hidden diversity for abiotic and biotic stress tolerances in the primary gene pool of rice revealed by a large backcross breeding program. Field Crops Research, 97, 66–76. 10.1016/j.fcr.2005.08.016

[tpg220189-bib-0003] Assaha, D. V. M. , Ueda, A. , Saneoka, H. , Al‐Yahyai, R. , & Yaish, M. W. (2017). The role of Na^+^ and K^+^ transporters in salt stress adaptation in glycophytes. Frontiers in Physiology, 8, 509. 10.3389/fphys.2017.00509 28769821 PMC5513949

[tpg220189-bib-0004] Auwera, G. A. , Carneiro, M. O. , Hartl, C. , Poplin, R. , Del Angel, G. , Levy‐Moonshine, A. , Jordan, T. , Shakir, K. , Roazen, D. , Thibault, J. , Banks, E. , Garimella, K. V. , Altshuler, D. , Gabriel, S. , & Depristo, M. A. (2013). From FastQ data to high‐confidence variant calls: The genome analysis toolkit best practices pipeline. Current Protocols in Bioinformatics, 43, 11.10.1–11.10.33. 10.1002/0471250953.bi1110s43 PMC4243306 25431634

[tpg220189-bib-0005] Backman, T. W. H. , & Girke, T. (2016). systemPipeR: NGS workflow and report generation environment. BMC Bioinformatics, 17, 388. 10.1186/s12859-016-1241-0 27650223 PMC5029110

[tpg220189-bib-0006] Bandelt, H. J. , Forster, P. , & Rohl, A. (1999). Median‐joining networks for inferring intraspecific phylogenies. Molecular Biology and Evolution, 16, 37–48. 10.1093/oxfordjournals.molbev.a026036 10331250

[tpg220189-bib-0007] Batayeva, D. , Labaco, B. , Ye, C. , Li, X. , Usenbekov, B. , Rysbekova, A. , Dyuskalieva, G. , Vergara, G. , Reinke, R. , & Leung, H. (2018). Genome‐wide association study of seedling stage salinity tolerance in temperate *japonica* rice germplasm. BMC Genetics, 19, 2. 10.1186/s12863-017-0590-7 29298667 PMC5753436

[tpg220189-bib-0008] Bonilla, P. , Mackell, D. , Deal, K. , & Gregorio, G. (2002). RFLP and SSLP mapping of salinity tolerance genes in chromosome 1 of rice (*Oryza sativa* L.) using recombinant inbred lines. Philippine Agricultural Scientist, 65, 68–76.

[tpg220189-bib-0009] Cha‐um, S. , Trakulyingcharoen, T. , Smitamana, P. , & Kirdmanee, C. (2009). Salt tolerance in two rice cultivars differing salt tolerant abilities in responses to iso‐osmotic stress. Australian Journal of Crop Science, 3, 221–230.

[tpg220189-bib-0010] Cha‐um, S. , Vejchasarn, P. , & Kirdmanee, C. (2007). An effective defensive response in Thai aromatic rice varieties (*Oryza sativa* L. spp. *indica*) to salinity. Journal of Crop Science and Biotechnology, 10, 257–264.

[tpg220189-bib-0011] Chokwiwatkul, R. , Tantipirom, N. , Khunpolwatatna, N. , Imyim, A. , Suriya‐aroonroj, D. , Buaboocha, T. , Pongpanich, M. , Comai, L. , & Chadchawan, S. (2017). Identification of genes involving in salt tolerance using GWAS data based on Na^+^ content in local Thai rice leaves. Genomics and Genetics, 10, 27–37. 10.14456/gag.2017.5

[tpg220189-bib-0012] Chutimanukul, P. , Kositsup, B. , Plaimas, K. , Siangliw, M. , & Toojinda, T. (2019). Effect of salt stress on antioxidant enzyme activity and hydrogen peroxide content in chromosome segment substitution line of ‘Khao Dawk Mali 105’ rice. Agriculture and Natural Resources, 53, 465–471.

[tpg220189-bib-0013] Coffman, W. R. , & Gomez, K. A. (1978). Parentage of IRRI [College, Laguna, Philippines; rice] crosses: IR1 ‐ IR10,000. International Rice Research Institute.

[tpg220189-bib-0014] Danecek, P. , Auton, A. , Abecasis, G. , Albers, C. A. , Banks, E. , Depristo, M. A. , Handsaker, R. E. , Lunter, G. , Marth, G. T. , Sherry, S. T. , Mcvean, G. , & Durbin, R. , & 1000 Genomes Project Analysis Group . (2011). The variant call format and VCFtools. Bioinformatics, 27, 2156–2158. 10.1093/bioinformatics/btr330 21653522 PMC3137218

[tpg220189-bib-0015] De Leon, T. B. , Linscombe, S. , Gregorio, G. , & Subudhi, P. K. (2015). Genetic variation in Southern USA rice genotypes for seedling salinity tolerance. Frontiers in Plant Science, 6, 374. 10.3389/fpls.2015.00374 26074937 PMC4444739

[tpg220189-bib-0016] Fischer, R. , & Maurer, R. (1978). Drought resistance in spring wheat cultivars. I. Grain yield responses. Australian Journal of Agricultural Research, 29, 897–912. 10.1071/AR9780897

[tpg220189-bib-0017] Ganie, S. A. , & Reddy, A. S. N. (2021). Stress‐induced changes in alternative splicing landscape in rice: Functional significance of splice isoforms in stress tolerance. Biology, 10, 309. 10.3390/biology10040309 33917813 PMC8068108

[tpg220189-bib-0018] Garrison, E. , & Marth, G. (2012). Haplotype‐based variant detection from short‐read sequencing. *arXiv:1207.3907*.

[tpg220189-bib-0019] Gerona, M. E. B. , Deocampo, M. P. , Egdane, J. A. , Ismail, A. M. , & Dionisio‐Sese, M. L. (2019). Physiological responses of contrasting rice genotypes to salt stress at reproductive stage. Rice Science, 26, 207–219. 10.1016/j.rsci.2019.05.001

[tpg220189-bib-0020] Ghomi, K. , Rabiei, B. , Sabouri, H. , & Sabouri, A. (2013). Mapping QTLs for traits related to salinity tolerance at seedling stage of rice (*Oryza sativa* L.): An agrigenomics study of an Iranian rice population. OMICS: A Journal of Integrative Biology, 17, 242–251. 10.1089/omi.2012.0097 23638881

[tpg220189-bib-0021] Gregorio, G. B. (1997). Tagging salinity tolerance genes in rice using amplified fragment length polymorphism (AFLP) (Doctoral dissertation, University of the Philippines Los Baños, Laguna, Philippines.

[tpg220189-bib-0022] Gregorio, G. , Senadhira, D. , & Mendoza, R. (1997). Screening rice for salinity tolerance (vol. 22). IRRI discussion paper series. International Rice Research Institute.

[tpg220189-bib-0023] Guo, J. , Sun, B. , He, H. , Zhang, Y. , Tian, H. , & Wang, B. (2021). Current understanding of bhlh transcription factors in plant abiotic stress tolerance. International Journal of Molecular Sciences, 22, 4921. 10.3390/ijms22094921 34066424 PMC8125693

[tpg220189-bib-0024] Hakim, M. A. , Juraimi, A. S. , Begum, M. , Hanafi, M. M. , Ismail, M. R. , & Selamat, A. (2010). Effect of salt stress on germination and early seedling growth of rice (*Oryza sativa* L.). African Journal of Biotechnology, 9, 1911–1918. 10.4314/ajb.v9i13

[tpg220189-bib-0025] Islam, F. , Wang, J. , Farooq, M. A. , Yang, C. , Jan, M. , Mwamba, T. M. , Hannan, F. , Xu, L. , & Zhou, W. (2019). Rice responses and tolerance to salt stress: Deciphering the physiological and molecular mechanisms of salinity adaptation. In M. Hasanuzzaman , M. Fujita , K. Nahar , & J. K. Biswas (Eds.), Advances in rice research for abiotic stress tolerance (pp. 791–819). Woodhead Publishing.

[tpg220189-bib-0026] Ito, Y. , Katsura, K. , Maruyama, K. , Taji, T. , Kobayashi, M. , Seki, M. , Shinozaki, K. , & Yamaguchi‐Shinozaki, K. (2006). Functional analysis of rice DREB1/CBF‐type transcription factors involved in cold‐responsive gene expression in transgenic rice. Plant & Cell Physiology, 47, 141–153. 10.1093/pcp/pci230 16284406

[tpg220189-bib-0027] Kaminaka, H. , Morita, S. , Tokumoto, M. , Masumura, T. , & Tanaka, K. (1999). Differential gene expressions of rice superoxide dismutase isoforms to oxidative and environmental stresses. Free Radical Research, 31, 219–225. 10.1080/10715769900301541 10694063

[tpg220189-bib-0028] Kanawapee, N. , Sanitchon, J. , Srihaban, P. , & Theerakulpisut, P. (2011). Genetic diversity analysis of rice cultivars (*Oryza sativa* L.) differing in salinity tolerance based on RAPD and SSR markers. Electronic Journal of Biotechnology, 14.

[tpg220189-bib-0029] Kanawapee, N. , Sanitchon, J. , Srihaban, P. , & Theerakulpisut, P. (2013). Physiological changes during development of rice (*Oryza sativa* L.) varieties differing in salt tolerance under saline field condition. Plant and Soil, 370, 89–101. 10.1007/s11104-013-1620-5

[tpg220189-bib-0030] Kanjoo, V. , Jearakongman, S. , Punyawaew, K. , Siangliw, J. L. , Siangliw, M. , Vanavichit, A. , & Toojinda, T. (2011). Co‐location of quantitative trait loci for drought and salinity tolerance in rice. Genomics and Genetics, 4, 126–138. 10.14456/tjg.2011.3

[tpg220189-bib-0031] Katawatin, R. , & Sukchan, S. (2012). Mapping soil salinity and soil erosion in Thailand with emphasis on computer‐assisted techniques. Pedologist, 55, 343–354. 10.18920/pedologist.55.3_343

[tpg220189-bib-0032] Koboldt, D. C. , Larson, D. E. , & Wilson, R. K. (2013). Using VarScan 2 for germline variant calling and somatic mutation detection. Current Protocols in Bioinformatics, 44, 15‐4.1–15.4.17. 10.1002/0471250953.bi1504s44 PMC4278659 25553206

[tpg220189-bib-0033] Krishnamurthy, S. L. , Pundir, P. , Warraich, A. S. , Rathor, S. , Lokeshkumar, B. M. , Singh, N. K. , & Sharma, P. C. (2020). Introgressed Saltol QTL lines improves the salinity tolerance in rice at seedling stage. Frontiers in Plant Science, 11, 833. 10.3389/fpls.2020.00833 32595689 PMC7300257

[tpg220189-bib-0034] Krishnamurthy, S. L. , Sharma, S. K. , Kumar, V. , Tiwari, S. , Batra, V. , & Singh, N. K. (2014). Assessment of genetic diversity in rice genotypes for salinity tolerance using *Saltol* markers of chromosome 1. Indian Journal of Genetics and Plant Breeding, 74, 243–247. 10.5958/0975-6906.2014.00167.9

[tpg220189-bib-0035] Lekklar, C. , Pongpanich, M. , Suriya‐Arunroj, D. , Chinpongpanich, A. , Tsai, H. , Comai, L. , Chadchawan, S. , & Buaboocha, T. (2019a). Genome‐wide association study for salinity tolerance at the flowering stage in a panel of rice accessions from Thailand. BMC Genomics, 20, 76. 10.1186/s12864-018-5317-2 30669971 PMC6343365

[tpg220189-bib-0036] Lekklar , C. , Suriya‐Arunroj , D. , Pongpanich , M. , Comai , L. , Kositsup , B. , Chadchawan , S. , & Buaboocha, T. (2019b). Comparative genomic analysis of rice with contrasting photosynthesis and grain production under salt stress. Genes, 10, 562. 10.3390/genes10080562 31349693 PMC6722916

[tpg220189-bib-0037] Lin, H. X. , Zhu, M. Z. , Yano, M. , Gao, J. P. , Liang, Z. W. , Su, W. A. , Hu, X. H. , Ren, Z. H. , & Chao, D. Y. (2004). QTLs for Na^+^ and K^+^ uptake of the shoots and roots controlling rice salt tolerance. Theoretical and Applied Genetics, 108, 253–260. 10.1007/s00122-003-1421-y 14513218

[tpg220189-bib-0038] Lisa, L. A. , Seraj, Z. I. , Fazle Elahi, C. M. , Das, K. C. , Biswas, K. , Islam, M. R. , Salam, M. A. , & Gomosta, A. R. (2004). Genetic variation in microsatellite DNA, physiology and morphology of coastal saline rice (*Oryza sativa* L.) landraces of Bangladesh. Plant and Soil, 263, 213–228. 10.1023/B:PLSO.0000047727.24160.f3

[tpg220189-bib-0039] Manohara, K. K. , Morajkar, S. , Shanbhag, Y. , Phadte, P. , & Singh, N. K. (2021). Haplotype analysis of *Saltol* QTL region in diverse landraces, wild rice and introgression lines of rice (*Oryza sativa* L.). Plant Genetic Resources: Characterization and Utilization, 19, 289–298. 10.1017/S1479262121000320

[tpg220189-bib-0040] Minh, B. Q. , Schmidt, H. A. , Chernomor, O. , Schrempf, D. , Woodhams, M. D. , Von Haeseler, A. , & Lanfear, R. (2020). IQ‐TREE 2: New models and efficient methods for phylogenetic inference in the genomic era. Molecular Biology and Evolution, 37, 1530–1534. 10.1093/molbev/msaa015 32011700 PMC7182206

[tpg220189-bib-0041] Mohammadi, R. , Mendioro, M. S. , Diaz, G. Q. , Gregorio, G. B. , & Singh, R. K. (2013). Mapping quantitative trait loci associated with yield and yield components under reproductive stage salinity stress in rice (*Oryza sativa* L.). Journal of Genetics, 92, 433–443. 10.1007/s12041-013-0285-4 24371165

[tpg220189-bib-0042] Moradi, F. , & Ismail, A. M. (2007). Responses of photosynthesis, chlorophyll fluorescence and ROS‐scavenging systems to salt stress during seedling and reproductive stages in rice. Annals of Botany, 99, 1161–1173. 10.1093/aob/mcm052 17428832 PMC3243573

[tpg220189-bib-0043] Naghashzadeh, M. (2014). Response of relative water content and cell membrane stability to mycorrhizal biofertilizer in maize. Electronic Journal of Biology, 10, 68–72.

[tpg220189-bib-0044] Ninsuwan, U. , Pornsirika, P. , Jundee, N. , & Puima, N. (2013). Identification of salt tolerance in Thai indigenous rice on the basis of the Na/K ratio and salt stress responses. Asian Journal of Plant Sciences, 12, 247–251. 10.3923/ajps.2013.247.251

[tpg220189-bib-0045] Nounjan, N. , Siangliw, J. L. , Toojinda, T. , Chadchawan, S. , & Theerakulpisut, P. (2016). Salt‐responsive mechanisms in chromosome segment substitution lines of rice (*Oryza sativa* L. cv. KDML105). Plant Physiology and Biochemistry, 103, 96–105. 10.1016/j.plaphy.2016.02.038 26986930

[tpg220189-bib-0046] Pandit, A. , Rai, V. , Bal, S. , Sinha, S. , Kumar, V. , Chauhan, M. , Gautam, R. K. , Singh, R. , Sharma, P. C. , Singh, A. K. , Gaikwad, K. , Sharma, T. R. , Mohapatra, T. , & Singh, N. K. (2010). Combining QTL mapping and transcriptome profiling of bulked RILs for identification of functional polymorphism for salt tolerance genes in rice (*Oryza sativa* L.). Molecular Genetics and Genomics, 284, 121–136. 10.1007/s00438-010-0551-6 20602115

[tpg220189-bib-0047] Park, S.‐I. , Kim, J.‐J. , Shin, S.‐Y. , Kim, Y.‐S. , & Yoon, H.‐S. (2020). *ASR* enhances environmental stress tolerance and improves grain yield by modulating stomatal closure in rice. Frontiers in Plant Science, 10, 1752. 10.3389/fpls.2019.01752 32117337 PMC7033646

[tpg220189-bib-0048] Pearson, G. A. , Ayers, A. D. , & Eberhard, D. L. (1966). Relative salt tolerance of rice during germination and early seedling development. Soil Science, 102, 151–156. 10.1097/00010694-196609000-00003

[tpg220189-bib-0049] Ponce, K. S. , Guo, L. , Leng, Y. , Meng, L. , & Ye, G. (2021). Advances in sensing, response and regulation mechanism of salt tolerance in rice. International Journal of Molecular Sciences, 22, 2254. 10.3390/ijms22052254 33668247 PMC7956267

[tpg220189-bib-0050] Pongprayoon, W. , Tisarum, R. , Theerawittaya, C. , & Cha‐Um, S. (2019). Evaluation and clustering on salt‐tolerant ability in rice genotypes (*Oryza sativa* L. subsp. indica) using multivariate physiological indices. Physiology and Molecular Biology of Plants, 25, 473–483. 10.1007/s12298-018-00636-2 30956429 PMC6419860

[tpg220189-bib-0051] Rajendran, K. , Tester, M. , & Roy, S. J. (2009). Quantifying the three main components of salinity tolerance in cereals. Plant, Cell & Environment, 32, 237–249. 10.1111/j.1365-3040.2008.01916.x 19054352

[tpg220189-bib-0052] Rasool, S. , Hameed, A. , Azooz, M. M. , Siddiqi, T. O. , & Ahmad, P. (2013). Salt stress: Causes, types and responses of plants. In P. Ahmad , M. Azooz , & M. Prasad (Eds.) Ecophysiology and responses of plants under salt stress (pp. 1–24). Springer. 10.1007/978-1-4614-4747-4_1

[tpg220189-bib-0053] Razzaque, S. , Elias, S. M. , Haque, T. , Biswas, S. , Jewel, G. M. N. A. , Rahman, S. , Weng, X. , Ismail, A. M. , Walia, H. , Juenger, T. E. , & Seraj, Z. I. (2019). Gene expression analysis associated with salt stress in a reciprocally crossed rice population. Scientific Reports, 9, 8249. 10.1038/s41598-019-44757-4 31160691 PMC6546764

[tpg220189-bib-0054] Reddy, I. N. B. L. , Kim, B.‐K. , Yoon, I.‐S. , Kim, K.‐H. , & Kwon, T.‐R. (2017). Salt tolerance in rice: Focus on mechanisms and approaches. Rice Science, 24, 123–144. 10.1016/j.rsci.2016.09.004

[tpg220189-bib-0055] Sade, N. , Galkin, E. , & Moshelion, M. (2015). Measuring Arabidopsis, tomato and barley leaf relative water content (RWC). Bio‐protocol, 5, e1451–e1451. 10.21769/BioProtoc.1451

[tpg220189-bib-0056] Shah, W. H. , Rasool, A. , Saleem, S. , Mushtaq, N. U. , Tahir, I. , Hakeem, K. R. , & Rehman, R. U. (2021). Understanding the integrated pathways and mechanisms of transporters, protein kinases, and transcription factors in plants under salt stress. International Journal of Genomics, 2021, 5578727. 10.1155/2021/5578727 33954166 PMC8057909

[tpg220189-bib-0057] Shobbar, M.‐S. , Azhari, O. , Shobbar, Z.‐S. , Niknam, V. , Askari, H. , Pessarakli, M. , & Ebrahimzadeh, H. (2012). Comparative analysis of some physiological responses of rice seedlings to cold, salt, and drought stresses. Journal of Plant Nutrition, 35, 1037–1052. 10.1080/01904167.2012.671407

[tpg220189-bib-0058] Singh, V. , Singh, A. P. , Bhadoria, J. , Giri, J. , Singh, J. T. V., V. , & Sharma, P. C. (2018). Differential expression of salt‐responsive genes to salinity stress in salt‐tolerant and salt‐sensitive rice (*Oryza sativa* L.) at seedling stage. Protoplasma, 255, 1667–1681. 10.1007/s00709-018-1257-6 29740721

[tpg220189-bib-0059] Sirault, X. R. R. , James, R. A. , & Furbank, R. T. (2009). A new screening method for osmotic component of salinity tolerance in cereals using infrared thermography. Functional Plant Biology, 36, 970–977. 10.1071/FP09182 32688708

[tpg220189-bib-0060] Thomson, M. J. , De Ocampo, M. , Egdane, J. , Rahman, M. A. , Sajise, A. G. , Adorada, D. L. , Tumimbang‐Raiz, E. , Blumwald, E. , Seraj, Z. I. , Singh, R. K. , Gregorio, G. B. , & Ismail, A. M. (2010). Characterizing the Saltol quantitative trait locus for salinity tolerance in rice. Rice, 3, 148–160. 10.1007/s12284-010-9053-8

[tpg220189-bib-0061] Tin, H. Q. , Loi, N. H. , Labarosa, S. J. E. , McNally, K. L. , McCouch, S. , & Kilian, B. (2021). Phenotypic response of farmer‐selected CWR‐derived rice lines to salt stress in the Mekong Delta. Crop Science, 61, 201–218. 10.1002/csc2.20354

[tpg220189-bib-0062] Udomchalothorn, T. , Plaimas, K. , Comai, L. , Buaboocha, T. , & Chadchawan, S. (2014). Molecular karyotyping and exome analysis of salt‐tolerant rice mutant from somaclonal variation. The Plant Genome, 7. 10.3835/plantgenome2014.04.0016

[tpg220189-bib-0063] Utsunomiya, Y. T. , Milanesi, M. , Utsunomiya, A. T. H. , Ajmone‐Marsan, P. , & Garcia, J. F. (2016). GHap: An R package for genome‐wide haplotyping. Bioinformatics, 32, 2861–2862. 10.1093/bioinformatics/btw356 27283951

[tpg220189-bib-0064] Waziri, A. , Kumar, P. , & Purty, R. S. (2016). *Saltol* QTL and their role in salinity tolerance in rice. Austin Journal of Biotechnology & Bioengineering, 3, 1067.

[tpg220189-bib-0065] Yadav, A. K. , Kumar, A. , Grover, N. , Ellur, R. K. , Krishnan, S. G. , Bollinedi, H. , Bhowmick, P. K. , Vinod, K. K. , Nagarajan, M. , Krishnamurthy, S. L. , & Singh, A. K. (2020). Marker aided introgression of ‘*Saltol*’, a major QTL for seedling stage salinity tolerance into an elite Basmati rice variety ‘Pusa Basmati 1509’. Scientific Reports, 10, 13877. 10.1038/s41598-020-70664-0 32887905 PMC7474085

[tpg220189-bib-0066] Yamane, K. , Kawasaki, M. , Taniguchi, M. , & Miyake, H. (2008). Correlation between chloroplast ultrastructure and chlorophyll fluorescence characteristics in the leaves of rice (*Oryza sativa* L.) grown under salinity. Plant Production Science, 11, 139–145. 10.1626/pps.11.139

